# Factors Associated with Subjective Well-Being of Chinese Adolescents Aged 10–15: Based on China Family Panel Studies

**DOI:** 10.3390/ijerph19126962

**Published:** 2022-06-07

**Authors:** Hong Shi, Hanfang Zhao, Zheng Ren, Minfu He, Yuyu Li, Yajiao Pu, Xiangrong Li, Shixun Wang, Li Cui, Jieyu Zhao, Hongjian Liu, Xiumin Zhang

**Affiliations:** 1 Department of Social Medicine and Health Management, School of Public Health, Jilin University, Changchun 130021, China; shihong21@mails.jlu.edu.cn (H.S.); zhaohf20@mails.jlu.edu.cn (H.Z.); renzheng20@mails.jlu.edu.cn (Z.R.); heminfu82@163.com (M.H.); yuyu20@mails.jlu.edu.cn (Y.L.); puyj20@mails.jlu.edu.cn (Y.P.); lxr19@mails.jlu.edu.cn (X.L.); wangsx21@mails.jlu.edu.cn (S.W.); cuili21@mails.jlu.edu.cn (L.C.); zhao1479721642@163.com (J.Z.); 2 Department of Epidemiology and Biostatistics, School of Public Health, Jilin University, Changchun 130021, China; hongjianliu63@jlu.edu.cn

**Keywords:** subjective well-being, associated factors, adolescents, Chinese

## Abstract

(1) Background: This study aimed to explore the factors associated with the subjective well-being (SWB) of Chinese adolescents from a more comprehensive perspective and to analyze the importance of its influencing factors. (2) Methods: Obtained from the China Family Panel Studies (CFPS) 2018, the research data involved 2316 adolescents aged 10–15. By using the Chi-square test, *t*-test and binary logistic regression, we investigated the associations of individual-, family- and community-level factors with SWB in Chinese adolescents. To explore the rank of the influencing factors of SWB, the random forest model was set up. (3) Results: In individual-level factors, girls, who were adolescents with higher academic performance and school satisfaction, with the habit of midday napping and reading books, and with higher self-esteem, had a higher SWB. In family-level factors, mothers living at home resulted in a higher SWB, while quarrelling with their parents led to low SWB. In community-level factors, adolescents with better social relationships, social trust and who were better at telling their troubles to others had a higher SWB. Based on the random forest model, the importance degree was ranked, and the top five were decided, including self-esteem (89.949), social relations (43.457), academic performance (31.971), school satisfaction (27.651) and quarrelling with parents (19.026). (4) Conclusions: Self-esteem, social relations, academic performance, school satisfaction and quarrelling with parents are all important variables that are related to the SWB of Chinese adolescents.

## 1. Introduction

Adolescence is a period of important and profound developmental changes [1], in which the self-consciousness of adolescents sees rapid development with the increase in physical and cognitive development [2]. People are increasingly realizing the importance of the social and emotional development of adolescents [3,4]. Promoting the social and emotional well-being of adolescents serves as an important determinant of their positive development, which enables them to achieve positive outcomes in school, work and life more generally [5].

Adolescence is a critical period characterized by some factors that can exert a significant influence on the subjective well-being (SWB) of adolescents. As the core notion of positive psychology, SWB refers to individuals’ cognitive and affective evaluations of their lives, a subjective global state of satisfaction and positive mental health [6]. Prior research has shown that SWB is closely related to individual development such as the psychological perception of people [7], which is a key index for evaluating the positive development and mental health of adolescents [8]. The SWB of adolescents shows a downward trend with age [9]. The levels of SWB witness a progressive decrease from early to late adolescence [1], which means that SWB may have a major effect on the positive results of the smooth transition of adolescents to adulthood.

It is necessary to understand the related factors first to effectively improve SWB. The ecological model articulated presumes that individual development occurs within the context of interconnected systems [10], which indicates that the factors affecting the SWB of adolescents must be multifaceted. Meanwhile, the ecological system theory shows that well-being is the product of interaction between ever-changing individuals and environments [11]. The environments that the adolescents can directly interact with, including their family, school and peer relationships, play an important role in their cognitive and affective development [12].

With the ecological model, it is necessary to focus on the demands of contemporary society for social well-being, quality of life, good living and well-being. The United Nations proposed a total of 17 sustainable development goals (SDGs) in 2015, which mainly reflect that there has been a shift towards the inclusion of broader societal issues regarding ecological issues [13,14,15].

At the individual level, previous studies have pointed out that certain sociodemographic factors, including gender and age, are associated with adolescents’ SWB [9,16]. As a personal psychological quality, self-esteem can be effectively improved through interventions, which is one of the strongest predictors of SWB [17]. It has been previously found that higher self-esteem can exert positive affect and life satisfaction, but lower self-esteem results in negative affect [18,19,20,21]. Considerations should be given to academic factors as learning is a part of life for adolescents. It has been shown in a previous study that academic performance is related to SWB, indicating that positive affect in the present can predict the academic performance of students [22]. Moreover, some results suggest that higher academic achievement is associated with higher SWB [23,24]. In addition to academic factors, behavioral factors and some activities are associated with SWB. People are paying increasing attention to physical exercise, a positive behavior whose healthy effect on SWB among different age groups draws inconsistent conclusions [25]. Culturally defined to be part of a healthy lifestyle, regular midday napping is provided by schools for Chinese children and adolescents [26,27], the effect of which on the SWB of Chinese adolescents has limited evidence. A previous study of people aged 18 and over showed that there was a positive correlation between reading books and positive emotions [28], but a relative lack of research has been conducted on the relationship between reading books and SWB among adolescents.

At the level of the family, a study of Spanish adolescents pointed out that families are the keys to their SWB [29]. The family environment, such as the frequency of family activities, serves as a positive predictor of the SWB of adolescents [30]. At the same time, parent–child communication as a protective factor is conducive to the life and school satisfaction and happiness of adolescents [31,32]. Moreover, some other family factors, such as a family’s socioeconomic status and the educational level of the parents are also considered to be related to the development of adolescents [33]. At the level of the community, evidence suggests that social connectedness is an important predictor of well-being in adolescence [34]. Positive interpersonal relationships tend to have a positive impact on the SWB of adolescents [2,35]. Good social relations are likely to facilitate perceived support from friends and communities, thereby exerting a positive influence on adolescents’ development.

In comparison with the research on the SWB of those who are college-aged and adults, the SWB of children and adolescents has received comparatively less attention [1,36]. Although previous studies have shown an interest in the factors influencing the SWB of adolescents, most of them have done so from a single perspective. There is a lack of studies simultaneously investigating different levels of related factors. The previous studies on adolescents’ SWB mentioned above focused on demographic characteristics, personality factors, behavioral lifestyle factors, family factors and interpersonal factors. However, there were still inconsistent conclusions in some research [25,36,37]. What is more is that the previous studies mostly used traditional methods such as logistic regression to analyze influencing factors, while high prediction accuracy was achieved by random forest analysis, a supervised machine learning algorithm [38]. Through the comprehensive evaluation mechanism, the random forest can identify important influencing factors from complex multi-factors and conduct quantitative analysis, which contributes to targeted intervention on the key factors of adolescents’ SWB.

Overall, illuminating what specific key factors might be encouraged to improve the SWB of adolescents from a more comprehensive perspective and using more representative samples are still needed. Therefore, this study utilizes data from a large national sample of 10–15 year olds in China, aiming at examining the individual-, family- and community-level factors associated with the SWB of adolescents from a more comprehensive perspective. Furthermore, the study uses a random forest model to analyze the importance of its influencing factors, so as to provide new evidence for the development of more targeted intervention strategies that can improve the SWB of adolescents.

## 2. Materials and Methods

### 2.1. Data source and Research Sample

The data of this study were obtained from the 2018 wave of China Family Panel Studies (CFPS), which is a nationwide and comprehensive longitudinal tracking survey carried out by the China Social Science Research Center (ISSS) at Peking University, and aiming to reflect the socioeconomic, educational, demographic and health changes in China. Communities, families and individuals are the three important levels of CFPS surveys [39], the first of which was conducted in 2010 and the 2018 wave of which provides the latest data available in this study. CFPS visited about 15,000 families, collected about 44,000 personal questionnaires and surveyed 95% of the total population in China. The 2018 wave of CFPS survey originally included 2607 adolescents aged 10 to 15 and among whom cases with all study variables marked as “unknown”, “inapplicable” or missing information were excluded. Thus, the final sample comprised 2316 adolescents aged 10–15 in this study. The information for respondents was collected from adolescents themselves. The written informed consent to participation was obtained from the parents of the minors included in this study.

### 2.2. Measures

#### 2.2.1. Dependent Variables

Subjective well-being (SWB) was measured by the survey question “How happy do you feel about yourself?” scores of which ranged from 0 (“very unhappy”) to 10 (“very happy”) [40]. In this study, we divided 0–6 into low SWB and 7–10 into high SWB. The method of directly collecting data on the SWB of respondents is reliable, effective, meaningful and comparable [41].

#### 2.2.2. Independent Variables

Individual-level factors included gender (boys or girls), age (10–15) and residence (rural or urban). Academic performance was evaluated by the survey question “How do you rate your studies?” response categories of which ranged between 1 (very dissatisfied) and 5 (very satisfied), and responses were grouped into dissatisfied (scores 1 and 2), ambivalent (score 3) and satisfied (scores 4 and 5). Academic pressure was assessed by the survey question “How do you feel about your academic pressure?” response categories of which ranged between 1 (no pressure) and 5 (a lot of pressure), and responses were grouped into low (scores 1 and 2), fair (score 3) and high pressure (scores 4 and 5). School satisfaction was measured by the survey question “Are you satisfied with your school?” scores of which ranged from 1 (very dissatisfied) to 5 (very satisfied), and responses were divided into dissatisfied (scores 1 and 2), ambivalent (score 3) and satisfied (scores 4 and 5). Physical exercise was evaluated based on the question “Last week, what is your frequency of doing exercise, including in physical education classes?” Participants exercising at least once a week were classified as those with 1 = yes and others with 0 = no. Midday napping was classified as no or yes. Reading books was assessed by the survey question “Did you read books in the past 12 months other than for the purpose of work and examination? (including e-books but excluding newspapers and magazines)” answers of which were divided into “no” or “yes”. Smoking and alcohol drinking are two aspects of behavioral lifestyle. However, the respondents of this study reported very low rates of smoking and alcohol drinking because of being adolescents aged 10–15. Therefore, smoking and alcohol drinking were not analyzed as influencing factors in this study.

The self-esteem level of adolescents was measured by Rosenberg Self-Esteem Scale (RSES), a self-rating scale consisting of five positive and five negative items [42]. All responses of the items were made on scales ranging from 1 (strongly disagree) to 5 (strongly agree). The total score is between 10 and 50. The researcher pointed out that the eighth item of the scale (“I hope I can win more respect for myself”) is not suitable for measuring the self-esteem of Chinese people [43]. Through performing tests, the Cronbach alpha with excluded and non-excluded item 8 was 0.690 and 0.623, respectively. Consequently, item 8 was deleted in this study. The final score range of self-esteem was 9–45 with higher scores representing higher levels of self-esteem.

Family-level factors included father’s educational level (primary school or below/middle school/high school/college degree or above), mother’s educational level (primary school or below/middle school/high school/college degree or above), household income (below 20,000/20,001–50,000/50,001–100,000/100,001–150,000/above 150,001 yuan), fathers living at home (no or yes), mothers living at home (no or yes), quarrelling with parents (no or yes) and heart-to-heart talks with parents (no or yes).

Community-level factors included social relations, social trust and telling troubles to others. Social relations were measured by the survey question “How popular do you think you are?” scores of which ranged from 0 (the lowest) to 10 (the highest) and were classified into two groups, namely 0–6 and 7–10, respectively. Social trust was measured by asking respondents the question “In general, do you think that most people are trustworthy or we must be careful when getting along with others?” answers of which were divided into “untrustworthy” or “trustworthy”. Telling troubles to others was measured by the survey question “Who will you tell to most when you encounter troubles?” Adolescents’ choice of talking to parents, brothers and sisters, grandparents, teachers, classmates, friends and psychological counselors was summarized as “confide in others”, while their choice of “not telling others” and “confiding in the diary” was summarized as “don’t confide in others”.

### 2.3. Statistical Analysis

Chi-square test and *t*-test were performed to analyze the differences in the SWB of Chinese adolescents with a variety of characteristics. Multivariate analysis was tested by binary logistic regression. The associated factors of the SWB among Chinese adolescents were further investigated through performing binary logistic regression and using the odd ratio (OR) and 95% confidence intervals (CIs). Before conducting binary logistic regression, independent variables were tested for collinearity according to the tolerance values and the VIF (variance inflation factor). The criterion values for tolerance and VIF (≤0.10 and ≥10, respectively) were adopted to identify collinearity [44]. Statistical analysis was carried out by using the Statistical Product and Service Solutions (SPSS) 24.0 (IBM Corp, Armonk, NY, USA).

The random forest model was used to further rank the importance of the variables with statistical significance in multivariate analysis. We measured the feature importance according to the Mean Decrease Gini involved in random forest algorithm, which indicates the purity of a dataset’s partition [45]. In this study, the outcome variable refers to the level of SWB (low/high) of adolescents, which is the ultimate purpose of decision making and classification. Explanatory variables were a series of influencing factors of SWB, which were used to classify outcome variables. We selected the parameters “ntree = 500”, “mtry = 3” to construct a random forest model, where approximately 70% (*n* = 1621) of the samples were selected to construct the training set and the rest 30% (*n* = 695) as the validation set. In addition, 5-fold cross-validation was adopted to implement the analysis. The statistical analysis was performed using randomForest package of R version 4.1.2 software.

## 3. Results

Table 1 shows the sample characteristics and factors associated with SWB among Chinese adolescents. The sample comprised 2316 Chinese adolescents aged between 10 and 15. Among them, 53.9% were boys and 58.7% were rural. Univariable analysis indicated that gender, residence, academic performance, school satisfaction, physical exercise, midday napping, reading books, self-esteem in individual-level factors, and father’s educational level, mother’s educational level, household income, mothers living at home, quarrelling with parents, heart-to-heart talks with parents in family-level factors, and social relations, social trust, telling troubles to others in community-level factors were all significantly associated with the SWB of adolescents (*p* < 0.05).

Table 2 shows the results of the collinearity analysis for this study. A collinearity analysis demonstrated a VIF < 10 and no collinearity among independent variables.

Table 3 displays the binary logistic regression results of the factors associated with the SWB and are identified as variables in the multivariate analysis if observed to be related to the SWB of Chinese adolescents in univariate analysis. After adjusting for age, in individual-level factors, girls had a higher SWB than boys. Adolescents satisfied with their academic performance and their school had a higher SWB. Midday napping habits and reading books contributed to a higher SWB of adolescents. In addition, the higher the self-esteem of adolescents was, the more likely they were to have a higher SWB. In family-level factors, the mothers living at home resulted in a higher SWB of adolescents, while those quarrelling with their parents led to a low SWB. In community-level factors, adolescents with better social relationships and social trust and able to tell their troubles to others had a higher SWB.

According to Mean Decrease Gini, the random forest’s variable importance is presented in Table 4. The importance of the factors influencing SWB ranged from high to low are: self-esteem (89.949), social relations (43.457), academic performance (31.971), school satisfaction (27.651), quarrelling with parents (19.026), gender (18.601), reading books (17.655), social trust (17.565), mothers living at home (16.868), telling troubles to others (15.982) and midday napping (15.762).

## 4. Discussion

Based on a large national sample of 10–15 year olds in China, this study explored the factors associated with the SWB of Chinese adolescents from a more comprehensive perspective. Furthermore, a random forest model was established to find out the importance of the influencing factors of SWB. In this study, a new finding is provided: that is, the top five important factors associated with SWB were self-esteem, social relations, academic performance, school satisfaction and quarrelling with parents. The results of this study contribute to a better understanding of how to increase the protective factors in adolescent life and lay a foundation to develop targeted measures to improve the SWB of adolescents.

Based on the random forest model, it is found that self-esteem in the individual-level factors was most significantly related to the SWB of adolescents. To be specific, higher self-esteem contributes to higher SWB, which supports the findings of a previous study [19]. At the same time, a systematic review showed that self-esteem is positively associated with SWB in most countries [18]. However, compared to collectivist societies, self-esteem plays a more central role in the determination of SWB in individualist societies. This is partly due to differences in the reliability and validity of measures and partly because of differences in cultural values and norms [46,47]. Terror Management Theory has put forward that self-esteem can encourage positive affect and psychological well-being [48]. Empirically, as an important personality variable, self-esteem has an influence on the cognition and emotions of people [49]. Individuals with a high level of self-esteem are more optimistic about the future and tend to see the positive side of things and evaluate their lives in a positive way to escalate their SWB. Moreover, people with high self-esteem may receive more positive feedback from their social environment, thus improving their SWB [50]. The interventions of group counseling, mental health education and curriculum training to increase the self-esteem levels of adolescents and then promote the improvement of their SWB are valuable.

Social relations in the community-level factors was the second most significantly related factor to adolescents’ SWB after self-esteem. The culture of collectivism emphasizes the interdependent self-concept and suggests that each group member is closely related to the other [51,52]. This study confirmed that social relationships were positively and significantly associated with SWB among Chinese adolescents, which is consistent with the previous research results about the SWB of Chinese adults [53]. Being successful in social relationships means fewer interpersonal troubles for adolescents. Meanwhile, positive social relations likely enable adolescents to obtain more resilient resources and promote positive outcomes. Adolescents with good social relations have social support networks, which enables them to feel a stronger sense of social belonging and further improves the level of SWB.

In this research, another beneficial result is that academic performance and school satisfaction are significantly related to adolescents’ SWB. Adolescence is both a turning point and a critical period for completing one’s studies. Adolescents who were satisfied with their academic performance had higher SWB, which is generally consistent with the results of the existing researches [24,54]. Chinese society places an extraordinarily high value on education [51,55]. Good academic performance is desired by adolescents themselves, parents, teachers and society in this sociocultural context. Through social comparison with peers, adolescents seek to affirm their sense of academic achievement. Moreover, a previous study, revealing a positive association between school grades and SWB, indicated that the learning outcomes can entail the positive result of boosting confidence and, ultimately, well-being [56]. The finding further showed that adolescents with higher school satisfaction had higher SWB. Schools play an important role in the life of students [4]. School satisfaction refers to students’ subjective cognitive evaluation of their school life, such as the campus environment, resource facilities and teacher–student relationships based on self-defined criteria [57]. Adolescents with higher school satisfaction are more likely to have a good material environment and positive interpersonal relationships that are beneficial to improving their SWB [56,58]. Thus, school personnel should keep abreast of students’ thoughts and feelings, create a favorable school environment, improve learning facilities and enhance students’ school satisfaction.

Family-level factors played an important role in predicting the SWB of Chinese adolescents. Previous classical studies have explored parenting styles associated with parental strictness or imposition and how they relate to adolescent well-being in ethnic minority families in the US, dangerous communities and collectivism Asiatic cultures such as China [59,60,61]. These studies recognized that parental strictness leads to optimal adjustment [59,60]. However, the results of this study showed that respondents quarrelling with parents had a lower SWB. Our study results are in line with a recent emergent study that raises serious questions about whether parental strictness is needed for children’s well-being in the current digital society [62]. It has clearly been shown that the greatest personal well-being was found for adolescents raised with lower parental strictness [62]. Furthermore, our study is also in line with recent research that conceptualized self-esteem as the valuative unidimensional part of a multidimensional self-concept [63,64], being well related to the optimum family in adolescent school adjustment [65], personal and social adjustment [66], and even families with children with an antisocial tendency [67]. Adolescents often have more conflicts with their parents. In addition, dealing with conflicts with negative methods such as bickering can affect the harmonious family atmosphere and, thus, the SWB of adolescents. Reducing or avoiding quarrelling with parents is beneficial to positive parent–adolescent relationships [68]. Positive parent–adolescent relationships, such as, in particular, important family support, promote the ability of adolescents to respond to challenges and pressures [69,70]. Taking into account that family cohesion serves as an important environmental resource for SWB [7], it is necessary for parents to pay attention to cultivating the perspective-taking ability of children [71]. The timely communication and effective interaction between parents and adolescents are [72] conducive to the expression of emotions, which can [73] promote the increase in positive emotions and the decrease in negative emotions, [74] further improving family cohesion. Moreover, our study did not find a significant association between household income and adolescents’ SWB. Additionally, previous research showed that a higher family socioeconomic status was not generally related to a higher SWB in the presence of family social capital [75]. This finding supports the claim that economic measures alone are not adequate to measure well-being, whereas the factors to promote adolescents’ SWB are positive parent–child relationships, loving care, understanding and a secure attachment [75]. For Chinese adolescents with a sense of family identity and interdependence among family members [7], parental support is crucial to improving their SWB.

This study also confirmed that despite their relatively low importance, gender, reading books, social trust, mothers living at home, telling troubles to others and midday napping were all good predictors of SWB. To be specific, a previous study on gender and SWB showed that girls had a higher mean of SWB than boys [76]. This is likely to be related to girls who showed higher values and better school adaptation than boys, thereby, in turn, improving their own SWB [76,77]. Social trust was positively correlated with the SWB of Chinese adults [78], which is consistent with our conclusion. Trust is associated with emotional support, and can improve SWB by facilitating social networks and support mechanisms. However, the relationship between social trust and SWB among adolescents is still worthy of exploration. The study showed that compared with their fathers living at home, their mothers living at home had a positive impact on the SWB of adolescents. In a previous study, the protective role of the mother–adolescent relationship for adolescents’ SWB was well-documented [79]. This can be explained by attachment theory, in which maternal attachment is conducive to promoting the psychological adjustment and SWB of adolescents [80]. Moreover, this study confirmed that midday napping had a positive impact on the SWB, thereby casting new light on the factors related to the SWB of adolescents, even if midday napping was the least significantly related factor to adolescents’ SWB among the 11 important variables. Sleeping is important to satisfy the basic needs of adolescents because of the physical and emotional development during adolescence [81]. Sleep shortage has not only been pervasive among adolescents but also is affected by a variety of factors. On the one hand, the use of electronic products, such as computers, telephones and the Internet, and longer screen times are associated with decreased sleep duration. On the other hand, excessive after-school activity and academic competitive stress are significantly linked to sleep shortage [82,83,84]. Moreover, a systematic review concluded that Asian adolescents’ bedtimes were later and sleep time was shorter than peers from North America and Europe [85]. In a previous study, the potential benefits of midday napping on various outcomes have been highlighted, indicating that midday napping is associated with reduced internalizing behavior problems, better academic achievement and greater happiness [27]. Meanwhile, routine midday napping may produce additional benefits for the heightened cognitive performance of adolescents [86]. Midday napping behavior can alleviate sleep shortage and ensure the best functioning of adolescents experiencing intensive learning and, in turn, increase their SWB.

Some studies have suggested that physical exercise is positively related to SWB [25,87], but our study did not find a significant association between physical exercise and the SWB of Chinese adolescents aged 10–15. Exercise motivation is an important guarantee factor for adolescents to participate in physical exercise [88]. It is possible that the Physical Education Examination of this group is a required course in China’s high school entrance examination, which requires this group to carry out certain physical exercise activities. The relationship between physical exercise and SWB needs to be further explored. Moreover, good exercise habits provide a more positive lifestyle, which is widely recognized. We recommend that adolescents’ long-term awareness of physical exercise should be cultivated.

## 5. Strengths and Limitations

### 5.1. Strengths

This study has some advantages. First, it utilized data from a large national sample of Chinese adolescents. Second, the individual-, family- and community-level factors associated with the SWB of adolescents were examined from a more comprehensive perspective, which is consistent with the ecological system theory. Third, the importance of influencing factors on adolescents’ SWB is further explored by using the random forest model, which extends our knowledge of the SWB for this population.

### 5.2. Limitations

Consideration should be given to the limitations of this study. Firstly, the use of cross-sectional data prevented the interpretation of causal relationships. Further confirmation and replication are required of the findings of this study as a significant influencing factors for adolescents’ SWB. In future research, it is necessary to examine the causal relationships among research variables by using longitudinal data. Secondly, subjective biases cannot be ruled out because of the self-reported information about SWB and related factors. This is likely to lead to biases in the process of information collection and an overestimation of the connection strength between SWB and some variables.

## 6. Conclusions

This study identified the factors related to the SWB of adolescents from the perspective of individual-, family- and community-level factors. The findings of this study confirmed that a total of 11 factors were related to Chinese adolescents’ SWB. Through the random forest model, the importance of the above 11 factors is ranked as follows: self-esteem, social relations, academic performance, school satisfaction, quarrelling with parents, gender, reading books, social trust, mothers living at home, telling troubles to others and midday napping. In determining SWB for adolescents, the individual, family, school and community factors were significant, which confirmed that the quality of adolescents’ relationships with their immediate environments matters. The study findings will serve to raise awareness and understanding among parents, educators and adolescents themselves of the factors associated with SWB. Specifically, adolescent groups with poor academic performance and low school satisfaction should receive more attention. Moreover, the SWB of the adolescent may be further facilitated by improving their self-esteem, social relations and parenthood. All of these results are likely to provide new empirical evidence for the development of more targeted intervention strategies that can improve the SWB of adolescents.

## Figures and Tables

**Table 1:** Univariable analysis of factors associated with subjective well-being (SWB) among Chinese adolescents (N = 2316).

Variables	Sample *n* (%)	Subjective Well-Being		*χ*^2^/*T*	*p*
		Low (*n* = 464)	High (*n* = 1852)		
**Individual-Level Factors**					
Gender				6.655	0.010
Boys	1249 (53.9)	275 (22.0)	974 (78.0)		
Girls	1067 (46.1)	189 (17.7)	878 (82.3)		
Age (year)				4.792	0.442
10	410 (17.7)	90 (22.0)	320 (78.0)		
11	404 (17.4)	72 (17.8)	332 (82.2)		
12	386 (16.7)	81 (21.0)	305 (79.0)		
13	420 (18.1)	91 (21.7)	329 (78.3)		
14	396 (17.1)	69 (17.4)	327 (82.6)		
15	300 (13.0)	61 (20.3)	239 (79.7)		
Residence				5.644	0.018
Rural	1360 (58.7)	295 (21.7)	1065 (78.3)		
Urban	956 (41.3)	169 (17.7)	787 (82.3)		
Academic performance				54.158	<0.001
Dissatisfied	271 (11.7)	88 (32.5)	183 (67.5)		
Ambivalent	1117 (48.2)	250 (22.4)	867 (77.6)		
Satisfied	928 (40.1)	126 (13.6)	802 (86.4)		
Academic pressure				1.132	0.568
Low	802 (34.6)	155 (19.3)	647 (80.7)		
Fair	851 (36.8)	167 (19.6)	684 (80.4)		
High	663 (28.6)	142 (21.4)	521 (78.6)		
School satisfaction				41.784	<0.001
Dissatisfied	159 (6.9)	53 (33.3)	106 (66.7)		
Ambivalent	383 (16.5)	107 (27.9)	276 (72.1)		
Satisfied	1774 (76.6)	304 (17.1)	1470 (82.9)		
Physical exercise				8.873	0.003
No	683 (29.5)	163 (23.9)	520 (76.1)		
Yes	1633 (70.5)	301 (18.4)	1332 (81.6)		
Midday napping				13.775	<0.001
No	1528 (66.0)	340 (22.3)	1188 (77.7)		
Yes	788 (34.0)	124 (15.7)	664 (84.3)		
Reading books				28.692	<0.001
No	412 (17.8)	122 (29.6)	290 (70.4)		
Yes	1904 (82.2)	342 (18.0)	1562 (82.0)		
Self-esteem	2316 (100.0)	32.48 ± 4.36	34.31 ± 3.99	−8.245	<0.001
**Family-Level Factors**					
Father’s educational level				8.716	0.033
Primary school or below	889 (38.4)	203 (22.8)	686 (77.2)		
Middle school	921 (39.8)	177 (19.2)	744 (80.8)		
High school	318 (13.7)	55 (17.3)	263 (82.7)		
College degree or above	188 (8.1)	29 (15.4)	159 (84.6)		
Mother’s educational level				17.572	0.001
Primary school or below	1092 (47.2)	247 (22.6)	845 (77.4)		
Middle school	828 (35.7)	166 (20.0)	662 (80.0)		
High school	238 (10.3)	33 (13.9)	205 (86.1)		
College degree or above	158 (6.8)	18 (11.4)	140 (88.6)		
Household income (yuan)				16.711	0.002
≤20,000	250 (10.8)	69 (27.6)	181 (72.4)		
20,001–50,000	716 (30.9)	150 (20.9)	566 (79.1)		
50,001–100,000	815 (35.2)	163 (20.0)	652 (80.0)		
100,001–150,000	272 (11.7)	42 (15.4)	230 (84.6)		
≥150,001	263 (11.4)	40 (15.2)	223 (84.8)		
Fathers living at home				1.473	0.225
No	501 (21.6)	110 (22.0)	391 (78.0)		
Yes	1815 (78.4)	354 (19.5)	1461 (80.5)		
Mothers living at home				24.616	<0.001
No	382 (16.5)	112 (29.3)	270 (70.7)		
Yes	1934 (83.5)	352 (18.2)	1582 (81.8)		
Quarrelling with parents				4.695	0.030
No	1468 (63.4)	274 (18.7)	1194 (81.3)		
Yes	848 (36.6)	190 (22.4)	658 (77.6)		
Heart-to-Heart talks with parents				16.876	<0.001
No	1276 (55.1)	295 (23.1)	981 (76.9)		
Yes	1040 (44.9)	169 (16.3)	871 (83.7)		
**Community-Level Factors**					
Social relations				205.926	<0.001
0–6	818 (35.3)	296 (36.2)	522 (63.8)		
7–10	1498 (64.7)	168 (11.2)	1330 (88.8)		
Social trust				27.070	<0.001
untrustworthy	791 (34.2)	206 (26.0)	585 (74.0)		
trustworthy	1525 (65.8)	258 (16.9)	1267 (83.1)		
Telling troubles to others				26.125	<0.001
Yes	1951 (84.2)	355 (18.2)	1596 (81.8)		
No	365 (15.8)	109 (29.9)	256 (70.1)		

**Table 2:** Collinearity analysis of factors that associated with subjective well-being (SWB) among Chinese adolescents (N = 2316).

Variables	Collinear Statistics		Variables	Collinear Statistics	
	Tolerance	VIF ^1^		Tolerance	VIF
Gender	0.972	1.028	Mother’s educational level	0.569	1.758
Residence	0.771	1.296	Household income	0.803	1.246
Academic performance	0.926	1.080	Mothers living at home	0.955	1.047
School satisfaction	0.966	1.035	Quarrelling with parents	0.941	1.062
Physical exercise	0.908	1.101	Heart-to-Heart talks with parents	0.887	1.128
Midday napping	0.968	1.033	Social relations	0.934	1.070
Reading books	0.924	1.083	Social trust	0.968	1.033
Self-esteem	0.908	1.101	Telling troubles to others	0.945	1.059
Father’s educational level	0.636	1.573			

^1^ VIF = variance inflation factor.

**Table 3:** Binary logistic regression analysis of factors associated with subjective well-being (SWB) among Chinese adolescents (N = 2316).

Variables	Unadjusted OR (95% CI)	Adjusted OR (95% CI) ^a^
**Individual-Level Factors**		
Gender		
Boys	1.000	1.000
Girls	1.302 (1.305, 1.640) *	1.305 (1.036, 1.642) *
Residence		
Rural	1.000	1.000
Urban	1.027 (0.794, 1.328)	1.027 (0.794, 1.327)
Academic performance		
Dissatisfied	1.000	1.000
Ambivalent	1.274 (0.922, 1.759)	1.274 (0.922, 1.759)
Satisfied	1.715 (1.206, 2.439) **	1.703 (1.196, 2.423) **
School satisfaction		
Dissatisfied	1.000	1.000
Ambivalent	1.006 (0.645, 1.569)	1.006 (0.645, 1.569)
Satisfied	1.704 (1.147, 2.532) **	1.682 (1.130, 2.503) *
Physical exercise		
No	1.000	1.000
Yes	1.047 (0.817, 1.342)	1.054 (0.821, 1.351)
Midday napping		
No	1.000	1.000
Yes	1.358 (1.059, 1.742) *	1.372 (1.068, 1.762) *
Reading books		
No	1.000	1.000
Yes	1.372 (1.039, 1.811) *	1.376 (1.042, 1.817) *
Self-esteem	1.074 (1.045, 1.104) ***	1.075 (1.045, 1.105) ***
**Family-Level Factors**		
Father’s educational level		
Primary school or below	1.000	1.000
Middle school	1.046 (0.804, 1.360)	1.042 (0.801, 1.356)
High school	1.038 (0.702, 1.536)	1.033 (0.698, 1.528)
College degree or above	0.732 (0.414, 1.295)	0.729 (0.412, 1.290)
Mother’s educational level		
Primary school or below	1.000	1.000
Middle school	1.060 (0.813, 1.381)	1.054 (0.808, 1.374)
High school	1.405 (0.877, 2.251)	1.401 (0.874, 2.245)
College degree or above	1.318 (0.671, 2.590)	1.304 (0.663, 2.565)
Household income (yuan)		
≤20, 000	1.000	1.000
20, 001–50, 000	1.210 (0.833, 1.757)	1.208 (0.831, 1.754)
50, 001–100, 000	1.174 (0.805, 1.713)	1.171 (0.802, 1.709)
100, 001–150, 000	1.519 (0.928, 2.489)	1.515 (0.925, 2.483)
≥150, 001	1.523 (0.908, 2.552)	1.527 (0.910, 2.561)
Mothers living at home		
No	1.000	1.000
Yes	1.584 (1.195, 2.100) **	1.587 (1.197, 2.104) **
Quarrelling with parents		
Yes	1.000	1.000
No	1.402 (1.105, 1.779) **	1.395 (1.099, 1.771) **
Heart-to-Heart talks with parents		
No	1.000	1.000
Yes	1.224 (0.960, 1.560)	1.224 (0.960, 1.561)
**Community-Level Factors**		
Social relations		
Low (0–6)	1.000	1.000
High (7–10)	3.708 (2.954, 4.654) ***	3.734 (2.971, 4.691) ***
Social trust		
untrustworthy	1.000	1.000
trustworthy	1.362 (1.081, 1.715) **	1.368 (1.086, 1.724) **
Telling troubles to others		
No	1.000	1.000
Yes	1.512 (1.137, 2.010) **	1.519 (1.142, 2.020) **

Note: ^a^ adjusted for age (year). *** *p* < 0.001; ** *p* < 0.01; * *p* < 0.05.

**Table 4:** The rank of importance of factors associated with subjective well-being (SWB) among Chinese adolescents in random forest model.

Rank	Variables	Mean Decrease Gini
1	Self-esteem	89.949
2	Social relations	43.457
3	Academic performance	31.971
4	School satisfaction	27.651
5	Quarrelling with parents	19.026
6	Gender	18.601
7	Reading books	17.655
8	Social trust	17.565
9	Mothers living at home	16.868
10	Telling troubles to others	15.982
11	Midday napping	15.762

## Data Availability

The data were released to the researchers without access to any personal data. Data access link: http://www.isss.pku.edu.cn/cfps/ (accessed on 27 March 2021).
